# Stem Cells in Musculoskeletal Regeneration: From Benchtop to Bedside

**DOI:** 10.1155/2016/8432314

**Published:** 2016-08-14

**Authors:** Jiabing Fan, Dong-An Wang, Haifeng Liu, Hongbin Fan, Fang Yang

**Affiliations:** ^1^Division of Advanced Prosthodontics, School of Dentistry, University of California, Los Angeles, CA 90095, USA; ^2^Division of Bioengineering, Nanyang Technological University, Singapore 637457; ^3^Key Laboratory for Biomechanics and Mechanobiology of Ministry of Education, School of Biological Science and Medical Engineering, Beihang University, Beijing 100191, China; ^4^Institute of Orthopedic Surgery, Xijing Hospital, The Fourth Military Medical University, Xi'an 710032, China; ^5^Department of Biomaterials, Radboud University Medical Centre, 6500 HB Nijmegen, Netherlands

The continuing existent challenges of conventional surgical approaches for musculoskeletal reconstruction have led to the favorite of stem cell-mediated treatments over the last decade [1]. However, the in-depth understanding of underlying therapeutic mechanisms, regulatory mechanisms of stem cell differentiation, interaction of stem cells and inductive factors/delivery vehicles, and evaluation criteria* in vitro* and* in vivo* are indispensable to the wide practice of stem cells-based therapeutics for functionally restoring the musculoskeletal tissues from bench to bedside [2]. We are pleased to present a special issue encompassing both basic and translational aspects of stem cells-related research and review work in musculoskeletal regeneration including cartilage, bone, ligament, spinal cord, and meniscus, aiming to blossom in the effective use of stem cells for clinical application.

The proper cell source is one of the determinant elements to the success of stem cells-based tissue regeneration [3]. Mesenchymal stem cells (MSCs) hold the high accessibility as well as capacity of self-renewal and multidifferentiation, representing an attractive cell source for musculoskeletal tissue engineering [3]. In this special issue, S. Morikawa et al. presented a comprehensive review work recapitulating fundamental biology of MSCs and highlighting that neural crest may be a new cell pool attributable to the harvest of MSCs in favor of craniofacial bone repair. And also S. Liu et al. reviewed a large number of literatures to discuss recent advances in meniscus tissue engineering using cell-based strategy, suggesting that scaffold-free cell self-assembly method is a potential approach to manufacture a functional meniscus graft with robust mechanical properties. Another review work conducted by Z. Deng et al. concluded that currently matrix-assisted autologous chondrocyte transplantation is an optimal approach to the repair of cartilage defects after comparing the efficacy and safety of various tissue engineering approaches through a systemic review and meta-analyses. This review also indicated that there are no enough studies collected to verify the efficacy of MSC-based treatment in cartilage repair.

Natural, synthetic, or nanoscale scaffolds, as delivery vehicles, have been widely employed to carrier stem cells or inductive factors (gene, protein, and DNA) for tissue repair [4]. However, it necessitates the further enhancement of scaffold in both conductivity and inductivity [4]. Q. Li et al. conducted a comparative assessment of how two calcium phosphate/collagen composite materials affect osteogenic differentiation of adipose-derived stem cells (ASCs) which are thought to be a promising cell source in bone tissue engineering, demonstrating the hydroxyapatite/*β*-tricalcium phosphate composite scaffold is a better stimulator for ASCs proliferation and osteogenesis. To overcome the worrisome complications likely caused by exogenous FDA-approved bone morphogenetic protein 2 (BMP-2) in bone repair, Q. Xie et al. developed a core-shell PEI (polyethylenimine)/pBMP2- (plasmid BMP2-) PLGA (poly(lactic-co-glycolic acid)) electrospun scaffold using both gene modification and coaxial electrospinning techniques, which was revealed to be capable of sustaining expression of BMP-2 as well as enhancing the osteogenic differentiation of periodontal ligament stem cells.

The ultimate goal of fundamental studies involving stem cells and musculoskeletal regeneration is to translate stem cells to clinical application [5]. In a research article, K. Yaghoobi et al. discovered that herbal drug,* Lavandula angustifolia*, significantly promoted human umbilical mesenchymal Wharton's jelly stem cells in the treatment of spinal cord injury created in Wistar rats. Additionally, Z. Kakabadze et al. who are working in several well-known research institutes around world carried out a pioneering phase 1 clinical trial using autologous human bone marrow stem cell transplantation to treat patients with spinal cord injury. Through evaluation of 18 patients after treatment, the therapeutic effects are encouraging in the recovery of spinal cord injury, but further improvements such as establishment of a standard of* in vivo* evaluation are still needed in future investigation. Together, these findings may provide an alternative approach to the use of stem cell-mediated therapy for the restoration of spinal cord injury that is still a challenge in the current clinical treatment.

Furthermore, L. Sun et al. performed a profound work in exploring the effect of mechanical stretch on proliferation and matrix formation of BMSCs and anterior cruciate ligament fibroblasts, two types of cell identified as major seed cells serving ligament reconstruction. The outcomes of this study are also reminiscent of mechanical property that may be emphasized in the following study of stem cells-mediated tissue engineering. Lastly, R. J. F. C. Amaral et al. reported that human blood collected with an anticoagulant of sodium citrate may yield higher amount of human platelet-rich plasma (PRP) and exert higher proliferation of MSCs. The interesting findings suggest PRP as a potential supplement to promote MSC proliferation and differentiation.

In summary, the cutting-edge review and research articles presented by experts in the field of orthopedic surgery, stem cells, and tissue engineering were collected to be published in this special issue, prospectively being a cornerstone to spur stem cell therapy to be applied to musculoskeletal regeneration in clinic.

*Jiabing Fan* *Jiabing Fan* *Dong-An Wang* *Dong-An Wang* *Haifeng Liu* *Haifeng Liu* *Hongbin Fan* *Hongbin Fan* *Fang Yang* *Fang Yang*
